# A semi-automatic method for extracting mitochondrial cristae characteristics from 3D focused ion beam scanning electron microscopy data

**DOI:** 10.1038/s42003-024-06045-4

**Published:** 2024-03-28

**Authors:** Chenhao Wang, Leif Østergaard, Stine Hasselholt, Jon Sporring

**Affiliations:** 1 Department of Computer Science, University of Copenhagen, Copenhagen, Denmark; 2 Center for Quantification of Imaging Data from MAX IV, Copenhagen, Denmark; 3 Department of Clinical Medicine, Aarhus University, Aarhus, Denmark; 4 Center of Functionally Integrative Neuroscience, Aarhus, Denmark

**Keywords:** Neuroscience, Image processing, Software

## Abstract

Mitochondria are the main suppliers of energy for cells and their bioenergetic function is regulated by *mitochondrial dynamics*: the constant changes in mitochondria size, shape, and cristae morphology to secure cell homeostasis. Although changes in mitochondrial function are implicated in a wide range of diseases, our understanding is challenged by a lack of reliable ways to extract spatial features from the cristae, the detailed visualization of which requires electron microscopy (EM). Here, we present a semi-automatic method for the segmentation, 3D reconstruction, and shape analysis of mitochondria, cristae, and intracristal spaces based on 2D EM images of the murine hippocampus. We show that our method provides a more accurate characterization of mitochondrial ultrastructure in 3D than common 2D approaches and propose an operational index of mitochondria’s internal organization. With an improved consistency of 3D shape analysis and a decrease in the workload needed for large-scale analysis, we speculate that this tool will help increase our understanding of mitochondrial dynamics in health and disease.

## Abstract (web summary)

We present a new method for the efficient segmentation and 3D shape analysis of mitochondrial inner components from FIBSEM images using multiplanar UNET and persistent homology.

## Introduction

**Fig. 1:**
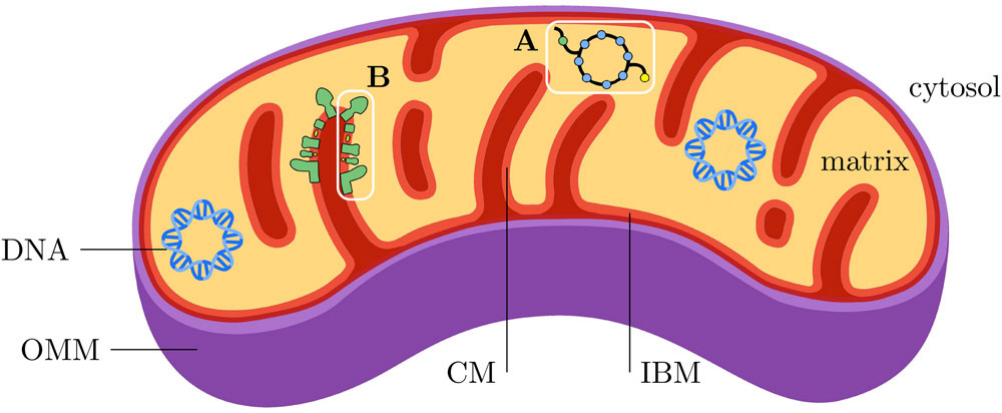
Structure of mitochondria. Mitochondria are characterized by two membranes that define three functional compartments. The outer mitochondrial membrane (OMM) acts as a barrier between the cytosol and the *intermembrane space*. The inner mitochondrial membrane (IMM), in turn, separates the intermembrane space from the *mitochondrial matrix*. In doing so, the IMM forms numerous invaginations into the matrix, the *mitochodrial cristae*. At the base of these cristae, the crista junction separates the IMM into crista membrane (CM) and inner boundary membrane (IBM), respectively, of which the latter runs largely parallel to the OMM, separated by the intermembrane space. The OMM is highly permeable to small solutes and contains proteins that allow larger molecules to pass. The IMM, however, acts as a tight diffusion barrier that only allows the passage of certain molecules via specific transport proteins. This enables the maintenance of a proton gradient between the intermembrane space and the matrix, which is critical for adenosine triphosphate (ATP) synthesis in the CM. While the matrix provides enzymes for citric acid cycle activity **A**, enabling the production of substrates for oxidative phosphorylation, the CM contains the respiratory chain protein complexes **B** that generate the ATP^29,60–62^. Graphics were in part produced by Vibe Fog Sporring.

Mitochondria are the main producers of adenosine triphosphate (ATP) via oxidative phosphorylation in eukaryotic cells. Consequently, the study of their function is relevant in a plethora of diseases. While functional analysis of mitochondria can be performed on fresh, untreated tissue^1,2^ it is not applicable to preserved specimens. To this end, mitochondrial function is thought to be closely linked to its structural characteristics. Specifically, the electron transport chain is located in the mitochondrial crista membrane (CM) (Fig. 1). Morphological features that may relate to function include (i) CM surface area, which we speculate could scale with the capacity for respiratory ATP production, and (ii) crista shape, as the assembly and stability of electron transport chain complexes depend on it^3^. In particular, high local CM curvature near complex V (ATP synthase) may facilitate ATP production^4–6^. Dimerized complex V imposes local curvature^4,7,8^, whereas loss of dimerization results in wider cristae with blunt apices^9^. In vitro, the degree of oligomerization was found to be higher in respiratory- than in glycolytic cells^6^. Methods to reliably extract cristae properties could thus potentially narrow the gap between structure and function.

The majority of previous and current studies demonstrating mitochondrial ultrastructure have used manual annotations on electron microscopical and -tomographical sections. This approach is labor intensive, typically restricting analyses to a limited number of mitochondria. Most often subsequent evaluation of crista properties has been performed in 2D^9–12^ and the validity of generalizations to 3D is questionable. Fortunately, studies of mitochondria and cristae in 3D based on manual reconstructions of individual mitochondria have also been performed for decades^13–17^. Mendelsohn et al. evaluated 12 mitochondria and within this small sample, the volume estimates varied by almost an order of magnitude^15^ highlighting the importance of analyzing a larger population to ensure a representative sample. This becomes even more important for the dynamic mitochondrial cristae^18–21^.

For large-scale analysis of mitochondria and cristae in 3D to be realistic, an automated approach is highly desirable. Recent advances in machine learning have made large-scale automatic segmentation of mitochondria feasible^22,23^ but until now, such automated tools have not allowed segmentation and analysis of the structures most intimately connected to mitochondrial function: the cristae. There may be several reasons for this. First, there is a scarcity of sufficiently large training datasets to train 3D segmentation models to identify cristae. Secondly, the distinction of cristae from other features in gray-scale images from the electron microscope is difficult. Thirdly, well-defined distance and curvature measures in 3D, that can be extracted automatically, are needed. In this work, we suggest solutions to the challenges, enabling large-scale analysis of mitochondria and their cristae in 3D based on the application of machine learning.

## Results

### Multiplanar UNet overcomes the 3D training set barrier

Supervised machine learning models require correctly labeled data points. For 3D segmentation models, the data points are image sub-volumes that need to be densely annotated by experts for proper model training and validation. This is a tedious process that involves the manual annotation of hundreds of consecutive image slices for each sub-volume, possibly introducing a directional bias since the annotator tends to label slice-wise in a single slicing direction. In contrast, 2D models work on image slices, which are considerably less labor-intensive to annotate, and models are often smaller and require less data to converge. However, the 3D structural information is lost.

**Fig. 2:**
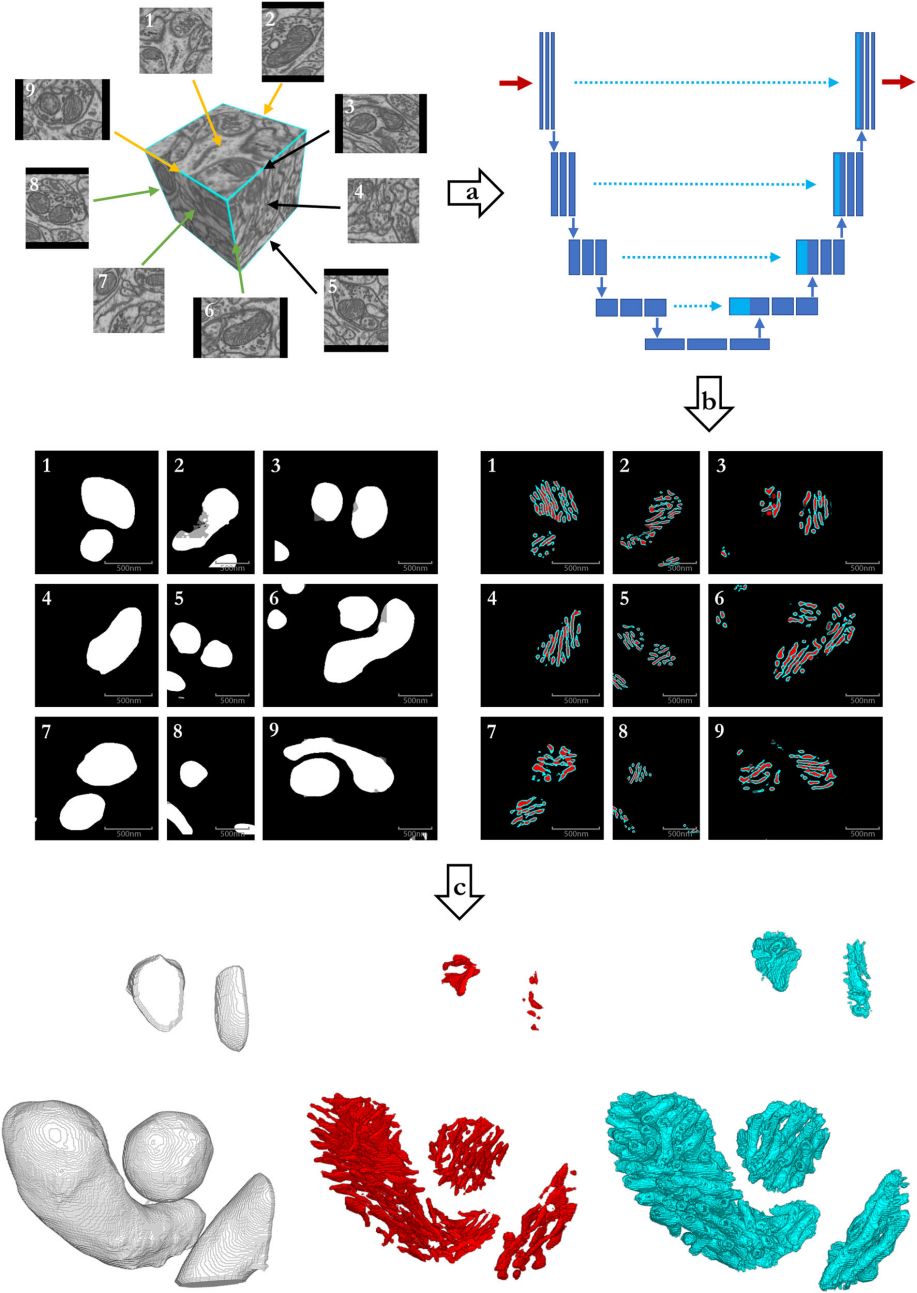
A Pipeline for segmenting cristae using the Multiplanar UNet. After drift is corrected by image registration, 2D image slices are extracted from nine different resliced planes. Selected image slices are annotated and fed to the UNet for training in step **a**. After training completes, the UNet is used in step **b** to segment the mitochondria (white), crista membrane (cyan), and intracristal space (red) in all nine planes. The resulting 2D stacks of segmentations from the different planes are combined using averaging, and cleaned up by mathematical morphology in step **c** to produce the final 3D segmentation (see Image Segmentation with the Multiplanar UNet). The segmentations produced by this pipeline form the basis for all subsequent analyses.

To get the best from both the 2D- and 3D models, we implemented a slightly modified version of the Multiplanar UNet^24^. This model is a 2D UNet^25^ that segments a 3D volume by merging 2D segmentations of images resliced in different orientations as shown in the top left of Fig. 2. At each voxel, the model produces several label candidates as a function of reslicing orientation, and these are merged by averaging the predicted softmax scores.

We incorporated the Multiplanar UNet in an active-learning approach to manual cristae annotation to facilitate the generation of new segmentation datasets. For details, see Image Segmentation with the Multiplanar UNet. The final segmentation workflow is illustrated in Fig. 2.

### Persistent homology allows distance and curvature measurements in 3D

**Fig. 3:**
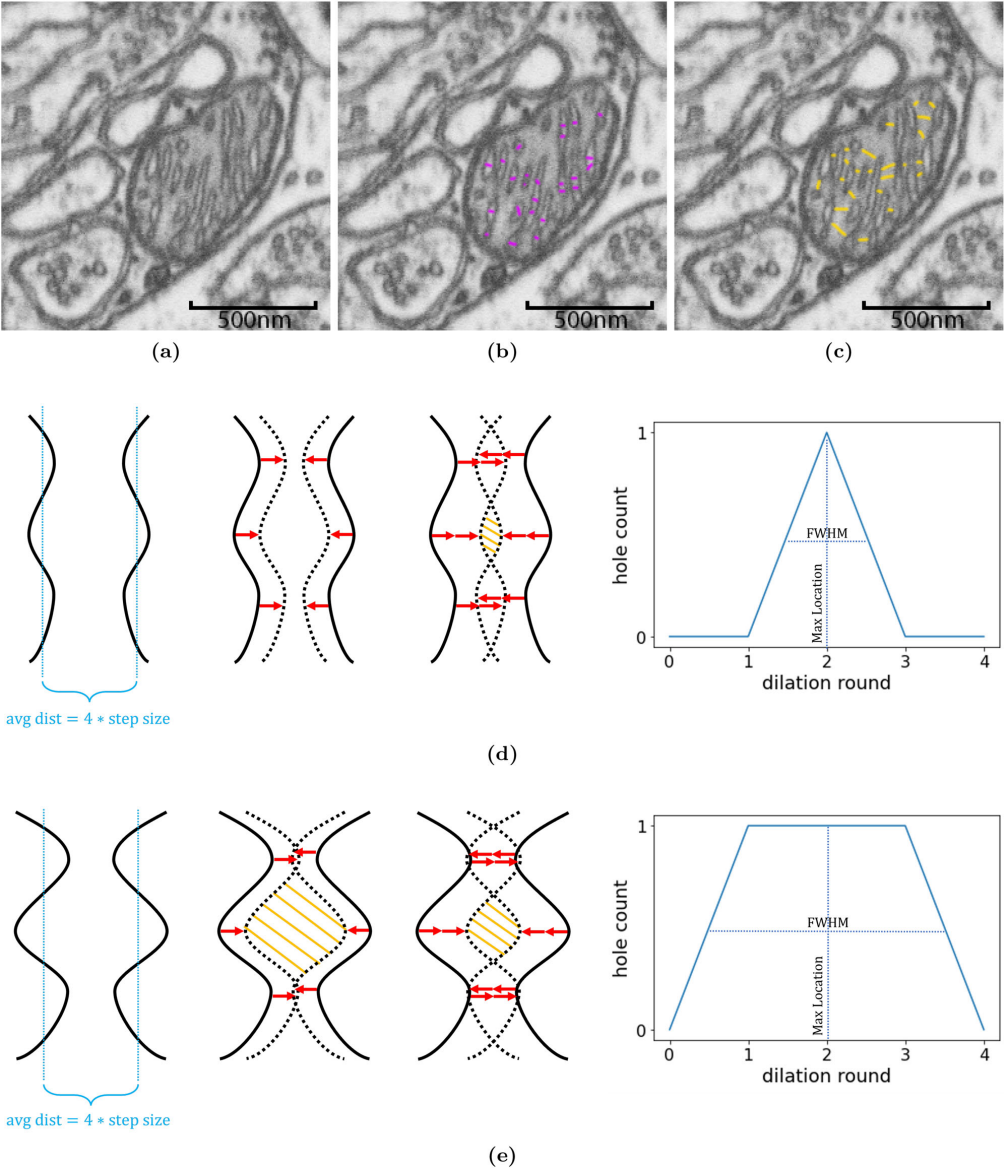
Persistent homology standardizes shortest distance measures in mitochondria. **a**–**c** Some of the many possible ways to measure crista widths (purple) and intercristal distances across matrix (yellow). **d**, **e** Persistent homology uses dilation and hole counting to standardize distance measurements. As pixels are added in dilation (red arrows), the crista membranes will move closer (dotted line) and a hole (yellow hatching) will form and eventually disappear. In crista membranes with a higher degree of curvature **e** holes will be present for more dilation rounds. This is indicated by a larger full-width-half-maximum in count curves generated during analysis, The max location in **d** and **e** remains the same at dilation round 2, which is equivalent to the average half distance between crista membranes.

Manual analyses of cristae and their organization poses several challenges. One is the lack of well-defined ways to measure relevant parameters. In the case of distance measurements, the choice of endpoints is not well-defined (see Fig. 3) making cross-study comparisons difficult. Even if clear definitions were available, minor deviations in adherence due to limitations in manual precision could have a large effect. Manual analyses are also often affected by 2D limitations, because while many software tools allow rotations of a 3D image volume, the interactive elements are designed in 2D for selection accuracy, which may introduce a measurement bias. Furthermore, a large number of measurements is needed for statistical validity, and this is not always feasible in manual analysis.

As a solution to these challenges, we used the concept of persistent homology to provide a standardized and directionally unbiased way to measure cristae distances and surface curvatures in 3-dimensional space. The idea behind persistent homology^26^ is to use features that exist and vary across a large parameter range to describe data that may be difficult to describe directly since their persistence is a sign of the real signal, rather than random effects from noise, sparseness or high dimensionality. In our case, the persistent features are the count curves of holes and objects that form as a function of the number of voxels being added to or removed from our segmentation surface by mathematical morphology (Fig. 3). Since morphology adds or removes voxels uniformly in all directions, the rate of hole or object formation depends solely on the shape of our segmentation. This means that it is possible to extract the features we need directly from the count curves, which we accomplish using the location of the maximum count (max location) and the full-width-half-maximum (FWHM). For more details on how this is done and the reasoning, please refer to Persistent Homology.

### Gross mitochondria morphology

**Fig. 4:**
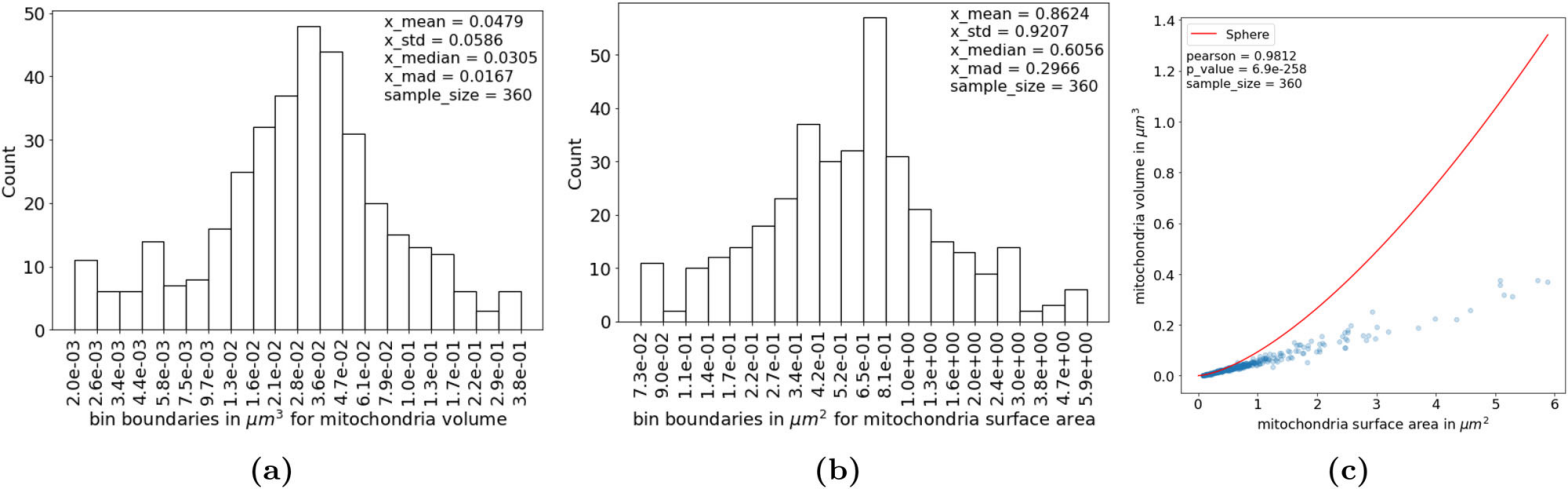
Morphology of mitochondria. The distribution of volumes **a** and surface areas **b** of mitochondria in 3D are shown for the full dataset. Please note that the counts and bin boundaries are computed on log-transformed values. The correlation between mitochondrial surface area and volume is almost perfectly linear. The curve represents the relationship between surface area and volume for a sphere **c**. mad median absolute deviation, std standard deviation.

For the mitochondria fully contained in the dataset, we found the mean volume to be 0.048 ± 0.059 μm^3^, where the uncertainty is given by the standard deviation (std). The median mitochondrial volume is lower at 0.031 ± 0.017 μm^3^, where the uncertainty is given as the median absolution deviation (mad). The mean mitochondrial surface area was found to be 0.86 ± 0.92 μm^2^, and the median mitochondrial surface area is again lower at 0.61 ± 0.30 μm^2^. Volume and surface area distributions are shown together with their correlation in Fig. 4. The mitochondrial volume and surface area show a near-perfect linear relationship with a Pearson correlation value of 0.98 (*p* = 6.9 × 10^−258^).

### Cristae morphology and organization

**Fig. 5:**
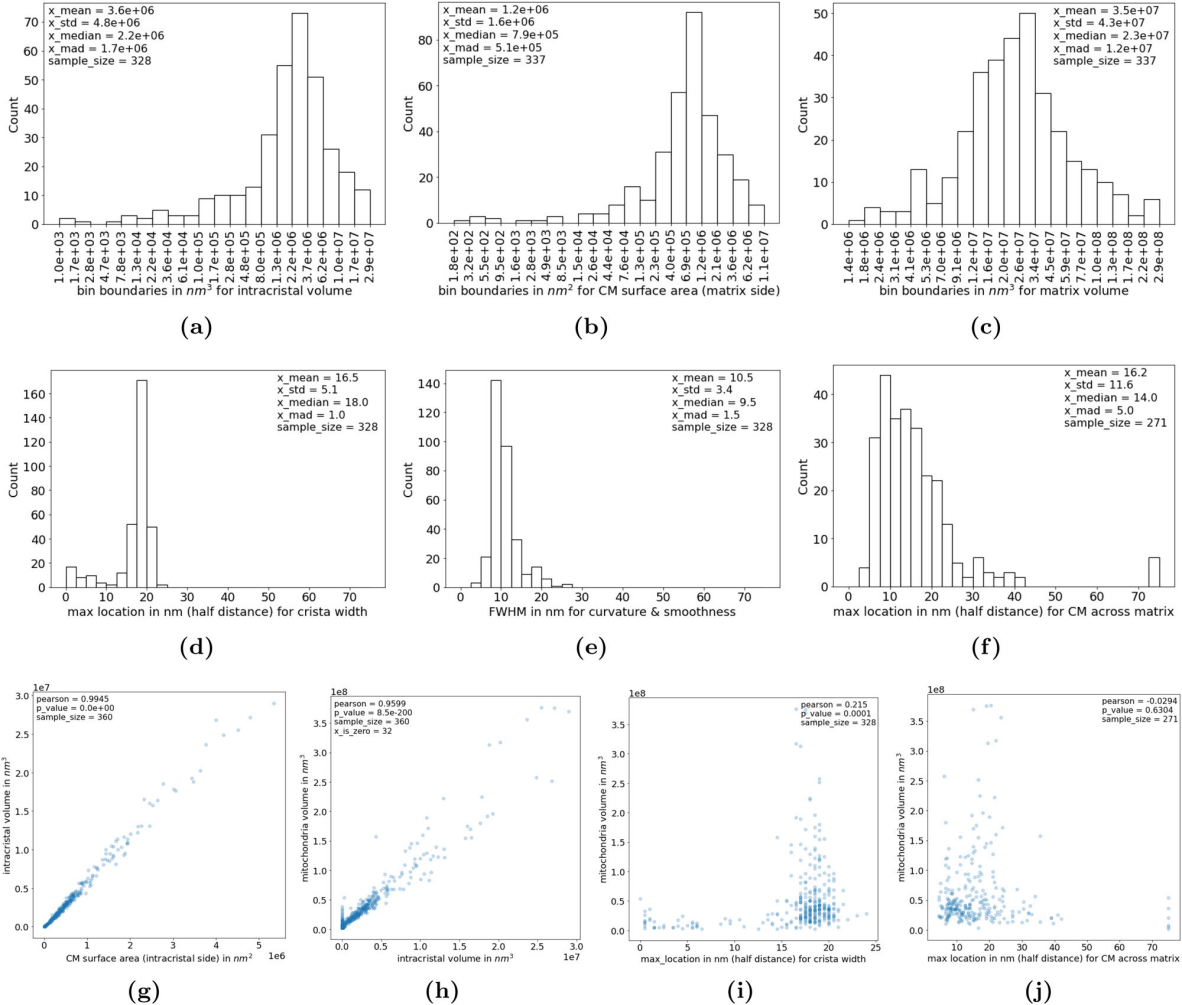
Morphology and organization of cristae. The distribution of total volumes of the intracristal space **a** and surface areas of mitochondrial cristae **b** in 3D are presented. Distributions of average crista half-widths **d**, cristae curvature/roughness **e**, and average half-distances between cristae across the matrix **f** in 3D are also shown. The distribution of volumes of the mitochondrial matrix is given in **c**. The correlations between cristae surface area (intracristal side) and intracristal volume are almost perfectly linear **g**. A positive linear relationship between the volumes of mitochondria and their intracristal spaces is also visible **h**. No clear relationship is detected between mitochondria volume and cristae width **i** or intercristal distance **j**. Please note that the counts and bin boundaries in histograms are computed on log-transformed values to better visualize the data, as the untransformed data are highly left skewed. For 32 out of 360 mitochondria, it was not possible to detect the inner mitochondria membrane. In subfigure **h** this results in the vertically scattered data points near the origin. Since the segmentation of these mitochondria does not seem to be erroneous, the data points are included in the scatter plot. The sample sizes vary for plots due to the differences in inclusion criteria for the parameters. CM crista membrane, FWHM full-width-half-maximum, mad median absolute deviation, std standard deviation.

The mean and median volumes of the intracristal space in murine hippocampal mitochondria were found to be 3.6 × 10^6^ ± 4.8 × 10^6^ nm^3^ and 2.2 × 10^6^ ± 1.7 × 10^6^ *nm*^3^, respectively (Fig. 5a). The mean and median CM surface area facing the matrix side was 1.2 × 10^6^ ± 1.6 × 10^6^ *nm*^2^ and 7.9 × 10^5^ ± 5.1 × 10^5^ nm^2^ (Fig. 5b).

The intracristal volume and the CM surface area (intracristal side) show a strong Pearson correlation of 0.99 with *p* = 0.0 × 10^0^ (Fig. 5g). We measured the crista width, defined here as the minimum distance across the intracristal space (CM included), using persistent homology as described in Persistent Homology. The mean and median crista widths were 33.0 ± 10.2 nm and 36.0 ± 2.0 nm, respectively (Fig. 5d). Another parameter extracted from persistent homology, the FWHM of the count curve, indirectly measures the relative smoothness and curvature of cristae. The mean and median FWHM are 10.5 ± 3.4 nm and 9.5 ± 1.5 nm (Fig. 5e). Generally, the smoother and less curved the CM is, the smaller the FWHM-value is, see Fig. 3 and Supplementary note 1.

The mean minimum distance between CM across the matrix is 32.4 ± 23.2 nm and the median is 28 ± 10.0 nm (Fig. 5f). This represents the average distance between individual cristae in murine hippocampal mitochondria.

A positive linear relationship between the volume of a mitochondrion and the volume of its intracristal space was seen in Fig. 5h (Pearson correlation of 0.96 (*p* = 8.5 × 10^−200^)). The volume of the mitochondrion, however, has no clear correlations with the width of its cristae and the distance between its cristae across the matrix (Fig. 5i, j). The mitochondrial matrix has a mean and median volume of 3.5 × 10^7^ ± 4.3 × 10^7^ nm^3^ and 2.3 × 10^7^ ± 1.2 × 10^7^ nm^3^ (Fig. 5c).

### Consistency between 2D and 3D mitochondria measures

Because the ultrastructure of mitochondria is frequently evaluated based on 2D EM images, we evaluated the correlation between 2D and 3D shape features from mitochondria in an attempt to assess the usability of 2D measurements. When comparing measures made on a single mitochondrion in 2D, the average error with respect to the 3D measure is around 86% for the parameters evaluated here, see Table 1. If a subset of 25, 50, or 100 mitochondria is measured in 2D, the average error of the mean is reduced to approximately 22%, 16 %, or 11%, respectively. Average errors for individual parameters can be found in Table 1 and the results of our correlation analysis are visualized in Supplementary Fig. 3. The variance of the expected 3D value, which is equivalent to the square of the error, changes approximately by a factor of $\frac{1}{n}$, as should be expected by the law of large numbers.

**Table 1:** The average expected error of a 3D parameter given a related 2D parameter estimated from a randomly chosen slice of a randomly chosen mitochondrion

Parameter	*n* = 1	*n* = 25	*n* = 50	*n* = 100
Mitochondrial Volume	85.01%	22.15%	15.71%	11.27%
Mitochondrial Surface Area	83.28%	19.49%	13.82%	9.97%
Intracristal Volume	86.10%	23.20%	17.13%	12.06%
Cristae Surface Area	88.12%	23.01%	15.92%	11.49%

Rows give the Mitochondrial and Intracristal Volume in 3D as compared to corresponding 2D sectional areas, and Mitochondrial and Cristal Surface Area in 3D as compared to corresponding 2D sectional circumference. Columns give the number of randomly selected mitochondria used for each estimate. E.g., when estimating the functional relation between the true Mitochondrial Volume *V* and the corresponding 2D area *A* from 25 randomly chosen mitochondria, we assume a conditional normal distribution *P*(*V*∣*A*) = *N*( *μ*_*V*_ (*A*), *σ*_*V*_ (*A*)) and estimate $\frac{{\sigma }_{V}(A)}{{\mu }_{V}(A)}=22.15 \%$. This means that an estimation of the average mitochondrial volume based on measurement of 2D cross-sectional area on a single random slice through each of 25 random mitochondria can be expected to be 22.15% erroneous. Please refer to 2D to 3D relationships for details and in particular Supplementary Fig. 3 which shows the corresponding *μ* and *σ* curves.

## Discussion

We have presented a pipeline enabling semi-automatic analysis of mitochondrial ultrastructure in 3D from a series of electron microscopice images. With a relatively small set of manually annotated images, automatic segmentation of the outer mitochondrial membrane, CM, and intracristal space from individual mitochondria was possible using the multiplanar 2D UNet. Subsequent estimation of 3D shape parameters for mitochondria and their cristae, along with an assessment of the distance between cristae in 3D, forms the basis for the evaluation of a population of mitochondria in a tissue of interest. The described methods are directly applicable to the study of conditions affecting mitochondria.

Using persistent homology, it is possible to acquire estimates for crista width (Fig. 5d) and relative curvature of the CM (Fig. 5e) in 3D. Local curvature of the CM has been proposed to enable proton up-concentration near complex V and to facilitate a kinetic coupling between the proton pumps and the ATP synthase, thereby increasing the possibility for the production of ATP^4–6^. In addition to the connection between complex V dimerization and crista width^9^, the width might also affect the diffusion distance for cytochrome c from the crista lumen to complex IV. Remodeling of mitochondrial cristae occurs as part of adaptive responses to altered energy substrate availability^3,27,28^ and during apoptosis^3,29,30^. A cell-type independent coupling between synaptic function, CM surface area, and crista shape has also been found^16^. Cristae change their configuration dynamically through elongation or shortening and detachment from or fusion with the inner boundary membrane^13,18^. They can also temporarily fuse to form networks^18–20^.

Persistent homology can also provide information about intercristal distance (Fig. 5f). A relatively homogenous intercristal distance could be important for keeping the respiratory chain at a short distance to required substrates including oxygen and adenosine diphosphate. For the mitochondria population in a sample, the mad on the intercristal distance indicates how well-organized the cristae are. A low mad indicates that the distance between individual cristae is relatively homogenous across mitochondria whereas a high mad indicates heterogeneity. Rajab et al. 2022 applied a semi-quantitative scoring system to evaluate this^31^. This is time-consuming, subjective, and requires a method for random selection of mitochondria to include in the analysis. The ratio between the intracristal volume and the mitochondrion volume also provides information about the organization of the mitochondrion. If small, it indicates that the mitochondrion has lost its inner characteristics i.e. cristae. In Fig. 5h, a vertical line of data points represents these mitochondria.

In addition to the evaluation of cristae, the assessment of gross mitochondrial morphology may give us a crude indication of tissue state. Mitochondrial morphology is modulated by cycles of fusion and fission events^32^ adapting the mitochondrial network to the availability of substrates and metabolic needs of the cell^27,33^. The selective fusion of mitochondria enables transfer/sharing of organelle components^32^ and allows for a more efficient energy conversion during substrate deficiency and acute oxidative stress^3,27,33^. Fission is involved in the removal of mitochondria^32^ and is a main event in apoptosis^34^. After fission, some mitochondria daughter organelles are depolarized targeting them for autophagy^32^. The shape of mitochondria is furthermore crucial for their proper axonal transport and distribution^35^. An indication of overall mitochondria shape is provided by the correlation between volume and surface area. In the population of mitochondria examined here, the relationship between mitochondrial volume and surface area is linear in accordance with a previous study of mouse cerebellum^15^. A curve representing the relationship between volume and surface area for a sphere has been added to the plot (Fig. 4). In vitro, elongated mitochondria have been shown to be spared during autophagy while more spherical mitochondria were not^27^. If the points in the correlation plot fall on the curve for a sphere, a plausible guess would therefore be that the mitochondria are damaged. The curve may also help in the evaluation of data reliability. A sphere has the smallest possible volume-to-surface area ratio so no points in the correlation plot should fall above the curve. We have explored whether the ratio between surface area and volume can be used as a standalone shape descriptor to distinguish if an object (e.g. the crista membrane) is lamellar or cylindrical, as previously done by others^15,16^. We concluded mathematically that it is not a good predictor. For more details, see Supplementary Note 2.

The frequent use of 2D EM images for evaluation of mitochondria and their cristae prompted us to make a comparison of the consistency between 2D and 3D measurements. We have already mentioned the reliability of results and analysis time as potential challenges related to 2D manual measurements (Introduction). An additional consideration relates to the characteristics of the tissue and region under study. If rotational invariance is present in the tissue, meaning that the distribution of size, orientation, and shape of objects is independent of the direction of imaging planes, then valid information can be obtained from 2D if enough images are analysed. In other words, if 2D images are acquired in a random orientation through a tissue, the mitochondria profiles available for analysis in the images should represent all sizes, orientations, and shapes of mitochondria in that tissue (see also fig. 10 in^36^). For many biological tissues, however, this is not the case and here it becomes important to use 3D shape for analysis as opposed to profiles from 2D slices.

From (Table 1) it can be seen that the average expected error of a 3D parameter based on a related 2D parameter depends on the number of random 2D profiles analysed. For a subset of 100 mitochondria, the 3D parameters evaluated in our dataset can be estimated based on 2D measurements with an average error (imprecision) of around 11%. If the average expected error in other datasets is similar, this means that for group comparisons, the mean difference between groups needs to be at least 22% to be detectable from 2D measurements. On top of this technical variation comes the biological variation. Whether a lower detection limit of 22% difference is sufficiently precise and biologically meaningful is study dependent. Further increasing the subset of mitochondria analysed will continue to decrease the error following a power law function. Simultaneously, there is an increase in the required workload and a need for very stringent rules to make measurements on different profiles consistent. Even then, it is our opinion that conclusions should be made carefully as minimal deviations in measurement precision can have a substantial impact on results, when the objects measured are as small as the mitochondrial cristae. Finally, if 2D measurements are made, a practical assessment of whether the requirement of rotational invariance is fulfilled is needed. From the evaluation of the distributions of shape parameters for mitochondria estimated in 3D in this study, we saw that the majority of the parameters have a mean that is significantly higher than the median. This suggests that a few extreme outliers are affecting the mean but due to the considerable sample size made feasible with semi-automatic detection, it is possible to identify them as outliers. A similar assessment in 2D would require a considerable number of manual annotations.

The 2D Multiplanar UNet, we have used here, has the advantage of needing only a limited amount of manually annotated images to train. It has a performance (Supplementary Note 3) comparable to current state-of-the-art 3D models for segmenting mitochondria^37^ and is less labor intensive. Various 3D models have been tested on mitochondria from the same dataset^22^, that was used here, and F1 scores between 0.901 and 0.947 were reported, against ours at 0.929 (supplementary table 2). For the segmentation of crista membrane and intracristal space, since as far as we know there is no densely annotated 3D volume available for testing, we cannot empirically evaluate the relative performance of the models. We speculate that they would be at similar level, with 3D models having a slight edge at the expense of needing exponentially more dense manual annotations in 3D. This is because 3D models can learn directly using an explicit view of the 3D structure, whereas 2D multiplanar models infer the 3D structure implicitly from multiple views. We suspect this may have a slightly larger effect on small structures like the crista membrane and intracristal space. Gross mitochondrial shape parameters in rodent brain^15,16,22^, and crista width based on manual reconstruction in rat- and chick nervous tissue, and Hela cells^13,14,18^, have previously been estimated in 3D. Our results (Figs. 4 and 5d) are in line with the previous findings. Our measure of intercristal distance (Fig. 5f) deviates from a previous finding in Hela cells (2D)^38^. It is unknown if the discrepancy stems from species- and tissue variation, methodological differences, or 2D to 3D discrepancy.

The internal organization of mitochondria is increasingly being evaluated, and focus on mitochondrial cristae changes in the study of disease has been seen in different research fields^10,31,39^. Cristae have been shown to increase in width and have more rounded apices under hypoxic conditions in vitro^40^ and in patients with the oxidative phosphorylation disease Leigh syndrome^9^.

Gomes et al. observed a connection between the elongation of mitochondria and increased cristae density during starvation (2D, in vitro and in vivo)^27^, altered organization of cristae have been shown in ovarian cancer (2D, in vitro)^41^, non-small cell lung cancer (2D, in vitro)^42^, and after cerebral ischemia (2D, in vitro)^10^, and ischemic stroke resulted in a loose, heterogeneous organization of cristae (2D, in vivo)^10^. The changes are most likely related to the pathological conditions as combining results about gross mitochondrial volume with information about internal distances in our sample suggests that the organization of the inner mitochondrial membrane is independent of mitochondria size (Fig. 5i, j), which suggest that pattern of cristae of comparable width is merely repeated more times in large mitochondria. Additional experiments are needed to determine if this is general for normal tissue. Even though there may be challenges with the interpretation of 2D analyses, the results by others presented here indicate the relevance of examining a spectrum of mitochondria of different sizes in disease and this is feasible with the methods described here.

A limitation to our analyses is that only mitochondria, where it is possible to segment cristae, can be included. The subpopulation where this is not possible may be of poorer quality due to organelle degradation, it may be an issue related to tissue processing, or a combination of the two. To evaluate the potential impact of this undesired selection, we suggest always assessing the fraction of the whole which these mitochondria constitute. In our study, 9% of the evaluated mitochondria were of this type. An additional challenge is that mitochondria gross shape parameters are affected by cellular location^13,23,43^. Separating mitochondria into subpopulations depending on location for example in the cell soma or in processes requires larger 3D volumes with concomitant increased imaging times and data amounts. Thirdly, individual mitochondria with touching membranes may be segmented as one. This potentially complicates the distinction between individual mitochondria and an interconnected network. However, with increasing sample size the impact of this error decreases. Moreover, it is always possible to go back and evaluate the raw images. Lastly, cristae shape is dependent on phylogenetic group^17,44–46^, species^46^, and tissue type^46^. Within the same tissue, the crista shape can vary with age^46^ and metabolic demand. In addition to natural shape variations, disease mediated changes in morphology occur. Our model was trained on mitochondria from the normal murine hippocampus. We expect the model to perform well on mitochondria with similar topology independent of origin. With markedly altered crista morphology, the requirement for ground truth data (i.e. manual annotations) and necessity of model retraining will increase.

For the future, we would like to find ways to reduce the workload for the end user even further, e.g., by exploring studies on uncertainty and diversity for annotation efficiency^37,47,48^. Another matter worth looking into is the segmentation performance when the mitochondria are more densely packed and touching. In our image volume, irrelevantly few mitochondria were inseparable, but the instantiation task may be more challenging for other datasets. In this case, modeling the outer and inner mitochondrial membranes separately may help. In addition, we are investigating whether the method can be successfully used on EM volumes with anisotropic voxels, i.e., differences in axial resolution, making the method compatible with a variety of imaging modalities. It is of great interest to apply our model to samples from different tissues, metabolic environments, and disease states to deepen our knowledge about the correlation between shape variations in cristae and mitochondria function. To strengthen the results from the ultrastructural analyses, they can be combined with an evaluation of mitochondrial function, e.g., via an analysis of glycolytic and aerobic metabolism^1^. Changes in ultrastructure may also be evaluated further using molecular biological analyses of important components in cristae assembly.

In conclusion, we provide a method for detailed analysis of mitochondrial ultrastructure in 3D based on a deep learning algorithm. From a limited amount of images with manually annotated ground truth data, we were able to reliably segment the mitochondria and their cristae. From these segmentations, we extracted information about crista surface area, volume, and shape. Furthermore, with the persistent homology method, introduced in this article, we derived statistical summary information about the internal organization of the cristae. Our new method is not restricted to cristae structures but can be applied to any other tubular shape.

## Methods

In this work, we have employed a standard deep-neural network for segmenting the images, estimated standard geometric object features, used a topology measure to characterize long-range object relations, and investigated the relationship between parameters estimated from 2D slices and measured in 3D, respectively. All of this will be detailed in the following.

The images used in this study were acquired by Graham Knott and Marco Cantoni from École Polytechnique Fédérale de Lausanne and are available from^49^: A 5-micrometer-cubed volume from *cornu ammonis* 1 in hippocampus from a mouse brain was imaged using Focused Ion Beam Scanning Electron Microscopy, and the mitochondria in two sub-volumes were annotated by experts. The image volume is 1065 × 2048 × 1536 voxel^3^, and the sub-volumes are 165 × 1024 × 768 voxel^3^. Voxel size is approximately 5 × 5 × 5 nanometer^3^. The original images show a small drift, hence, we performed image registration using vesicle-based drift correction^50^. Examples of the data together with the initial segmentation of mitochondria are shown in Fig. 2. To access the registered images and the annotated sub-volumes, please refer to Data availability.

### Image segmentation with the multiplanar UNet

All segmentations in this study were done using a modified version of the Multiplanar UNet^24^. The idea is that by merging 2D segmentation results from multiple planes at different angles, we can compensate for the loss of 3D information in 2D because structures not seen in one plane will most likely be visible in another plane. Further, since the core segmentation model is 2D, we can avoid having to annotate the ground truth masks in 3D, which is a labor-intensive task. To maximize the field of view for better 3D coverage, while minimizing the number of planes for computational efficiency, we used nine planes angled at 45 degrees to each other (Fig. 2).

We extended the original annotation of mitochondria in the 2 sub-volumes supplied by^49^ with our annotation of the CM and intracristal space. We used an active learning approach, iterating the following steps to build up a dataset of 150 image slices: Step 1 - Select a few 256 × 256 pixel image slices from the image volume. In the first iteration, this would be random slices from random planes. In all subsequent iterations, the slices can either be random (like the first iteration) or be chosen from places where the model didn’t work very well; Step 2 - Manually label or correct the CM in the cyan channel and the intracristal space enclosed by them in the red channel; Step 3 - Train a multi-class multiplanar 2D UNet, initialized with weights from the mitochondria segmentation task for transfer learning; Step 4 - Apply the multi-class multiplanar 2D UNet in a multi-planar fashion to acquire 3D results. The merging of results from different planes is achieved by averaging the predicted softmax scores; Step 5 - Repeat all steps until segmentation quality is sufficiently good.

The final model for CM and intracristal space was trained on the 150 selected slices chosen using the multiplanar active learning approach described above, where 80% was used for training and 20% for validation. Image augmentation consisting of translation, rotation, shearing, intensity adjustment, and noise introduction was actively applied to the training set. The augmentation parameters were chosen after extensive visual inspection of examples to make sure they are realistic and possible. The validation set was also augmented but only once to ensure each epoch is validated on the same data. The validation set also excluded intensity and noise augmentations to ensure we only validate on variations of real images without synthetic intensity values. Model optimization was done using the ADAM optimizer on Intersection over Union (IOU) loss. This is important because of the extremely unbalanced class ratio in favor of the background. To achieve efficient training speed and optimize performance, an adaptive learning rate was used, where we reduced the learning rate by a factor of 3 if the validation loss did not improve for 5 epochs, starting with learning rate 0.0001. The training was stopped early if the validation loss did not decrease for 25 epochs.

The segmentation results were binarized and cleaned up before performing any analysis. This included filling in the holes between the intracristal space and crista membrane by a maximum size threshold and binary openings to remove segmentation noise for the mitochondria. Mitochondria touching the boundary of the image volume, and mitochondria with a volume smaller than 25^3^ voxel^3^, were excluded from the analysis. The performance of our segmentation model was evaluated using 5-fold cross-validation (see Supplementary Note 3 and Supplementary Fig. 2).

The segmentation model can be extended to other datasets by repeating the active learning procedure described above either by fine-tuning or re-training from scratch as the user sees fit.

### Computation of basic shape measures

All shape measures were computed on a mitochondrion by mitochondrion basis and the results were collected as distributions of values. To link individual mitochondria with their respective CM and intracristal spaces, the mask from connected-component analysis on the mitochondria segmentation was multiplied with the segmentation of CM and intracristal space, respectively.

The volume is a measure of object size. Since our segmentation results are binary images, where the foreground is 1 and the background is 0, the volume is a summation of every voxel value (assuming the object has been isolated).

The volume unit can be converted to real-world measurement by multiplying with the image resolution (here 125 nm^3^). In this study, we measured the mitochondrion volume, the intracristal space volume, and the matrix volume, the latter of which is defined as:

1 $${{\mbox{matrix}}}{{\mbox{\_}}}{{\mbox{volume}}}= {{\mbox{mitochondrion}}}{{\mbox{\_}}}{{\mbox{volume}}}-{{\mbox{cristae}}}{{\mbox{\_}}}{{\mbox{membrane}}}{{\mbox{\_}}}{{\mbox{volume}}} \\ -{{\mbox{intracristal}}}{{\mbox{\_}}}{{\mbox{space}}}{{\mbox{\_}}}{{\mbox{volume}}}\,$$

for each object.

To calculate the surface area of an object, the segmentation volume was initially converted to a triangular mesh using marching cubes. The output of the algorithm contains a list of all vertices (with coordinate values) and all the triangular faces (made up of the vertex indices). The total surface area of the object is then the sum of the surface areas of the faces:

2 $$\,{{\mbox{surface}}}{{\mbox{\_}}}{{\mbox{area}}}={{\mbox{sum}}}{{\mbox{\_}}}{{\mbox{of}}}{{\mbox{\_}}}{{\mbox{face}}}{{\mbox{\_}}}{{\mbox{areas}}}({{\mbox{marching}}}{{\mbox{\_}}}{{\mbox{cubes}}}({{\mbox{object}}}\,))$$

In this study, we measured the surface areas of the mitochondria, the intracristal space, and the CM (facing the matrix).

### Persistent homology

In this paper, we combined persistent homology and image analysis. By applying mathematical morphology to our segmentation masks and counting the number of holes or objects that appear and disappear, we indirectly measured object distances and relative curvatures in 3-dimensional space.

Existing implementations of mathematical-morphology-based persistent homology are primarily designed to describe the distribution of multi-dimensional point clouds. One study^51^ did implement a method for calculating persistent homology of 3D image volumes at subpixel accuracy. Here, we extend their analysis with the notion of full-width half-maximum as an estimate of fine-scale curvature and present an alternative way of computing persistent homology, which produces a more suitable result structure for extracting novel and more complex features.

The two main types of morphological operations used in our persistent homology analysis were dilation and erosion which respectively enlarge or shrink an object uniformly in all directions by adding or removing a layer of voxels from the object’s surface. To enable measurements at subpixel accuracy, we took a PDE approach to mathematical morphology. Starting with the 2D first-order Osher-Sethian upwind scheme^52–55^, we adapt them to 3D by introducing new terms into the square root and subsequently enforcing a value constraint between 0 and 1:

Dilation

3 $${U}_{i,j,k}^{n+1}=	\,{U}_{i,j,k}^{n}+\lambda \left({\left(\max \left(0,{U}_{i+1,j,k}^{n}-{U}_{i,j,k}^{n}\right)\right)}^{2}+{\left(\max \left(0,{U}_{i-1,j,k}^{n}-{U}_{i,j,k}^{n}\right)\right)}^{2}\right.\\ 	+{\left(\max \left(0,{U}_{i,j+1,k}^{n}-{U}_{i,j,k}^{n}\right)\right)}^{2}+{\left(\max \left(0,{U}_{i,j-1,k}^{n}-{U}_{i,j,k}^{n}\right)\right)}^{2}\\ 	+{\left(\max \left(0,{U}_{i,j,k+1}^{n}-{U}_{i,j,k}^{n}\right)\right)}^{2}+{\left.{\left(\max \left(0,{U}_{i,j,k-1}^{n}-{U}_{i,j,k}^{n}\right)\right)}^{2}\right)}^{\frac{1}{2}}$$

4 $${U}_{i,j,k}^{n+1}=\left\{\begin{array}{ll}0,\quad &\,{{\mbox{if}}}\,\,{U}_{i,j,k}^{n+1} \, < \, 0\\ 1,\quad &\,{{\mbox{if}}}\,\,{U}_{i,j,k}^{n+1} \, > \, 1\\ {U}_{i,j,k}^{n+1},\quad &\,{{\mbox{else}}}\,\end{array}\right.$$

Erosion

5 $${U}_{i,j,k}^{n+1}=	\,{U}_{i,j,k}^{n}-\lambda \left({\left(\max \left(0,{U}_{i,j,k}^{n}-{U}_{i+1,j,k}^{n}\right)\right)}^{2}+{\left(\max \left(0,{U}_{i,j,k}^{n}-{U}_{i-1,j,k}^{n}\right)\right)}^{2}\right.\\ 	+{\left(\max \left(0,{U}_{i,j,k}^{n}-{U}_{i,j+1,k}^{n}\right)\right)}^{2}+{\left(\max \left(0,{U}_{i,j,k}^{n}-{U}_{i,j-1,k}^{n}\right)\right)}^{2}\\ 	+{\left.{\left(\max \left(0,{U}_{i,j,k}^{n}-{U}_{i,j,k+1}^{n}\right)\right)}^{2}+{\left(\max \left(0,{U}_{i,j,k}^{n}-{U}_{i,j,k-1}^{n}\right)\right)}^{2}\right)}^{\frac{1}{2}}$$

6 $${U}_{i,j,k}^{n+1}=\left\{\begin{array}{ll}0,\quad &\,{{\mbox{if}}}\,\,{U}_{i,j,k}^{n+1} \, < \, 0\\ 1,\quad &\,{{\mbox{if}}}\,\,{U}_{i,j,k}^{n+1} \, > \, 1\\ {U}_{i,j,k}^{n+1},\quad &\,{{\mbox{else}}}\,\end{array}\right.$$

Where *U* ^*n*^ represents the segmentation volume at the current timestep, *U* ^*n*+1^ represents the segmentation volume after a single round of subpixel morphology is applied to *U* ^*n*^, and *λ* represents the timestep. In our case, *λ* = 0.1 were used. This means that 10 rounds of subpixel morphology was be equivalent to one full round of standard mathematical morphology, and it represents the point where a single layer of voxels is either added or removed from the segmentation surfaces (depending on whether it is dilation or erosion). We applied persistent homology on a mitochondrion-by-mitochondrion basis.

Conceptually, in the case of dilation: As segmented objects are enlarged, previously non-touching points on different objects or different branches of the same object will eventually make contact with each other. When this happens, holes will begin to form in the background region and the hole count increases, see Fig. 3. As the dilation continues, different objects or different branches of the same object will fully merge and the holes that formed earlier will disappear. The resulting count curve, therefore, acts as an indirect shape descriptor from which information can be extracted. The equivalent curve can be obtained by erosion and object counting.

To measure the average minimum distance between cristae across the matrix, we performed dilation and hole counting on the sum of intracristal space- and CM segmentations (e.g. red objects + cyan objects in Fig. 2). For crista width, we also used the sum of intracristal space- and CM segmentation but performed erosion and object counting instead.

In all cases, each round of subpixel morphology produced a non-binary grayscale mask valued between 0 and 1. Although this raw mask is always the one to be used for the next round of subpixel morphology, a binarized version with a threshold of 0.5 was needed for the counting step. For hole counting after dilation, we first multiplied the binarized dilation mask with the mitochondrion segmentation to ensure the dilation did not go out of bounds. The dilation mask was then inverted before a 26-connected 3D-connected component algorithm was used to calculate the number of holes. Since an extra hole will always exist in the background, we subtracted 1 from the resulting count. Object counting after erosion works the same way, except counted without multiplying and inverting the mask, and we did not subtract anything from the object count.

We summarized our curves using the max location and FWHM, as measured by the iteration number. Given that the data was discrete and has a degree of randomness, the raw count curves will be slightly jagged and should be filtered using a Gaussian kernel. Due to noise susceptibility, the initial five rounds of subpixel morphology, corresponding to half of a full round of dilation/erosion, were not included when finding the max locations and their FWHM.

The max location can be interpreted as half of the average minimum distance across the region being dilated or eroded because an iteration having the most holes/objects implies that it is also the iteration where most surface points make contact. The FWHM measures the surface smoothness and curvature of the same region. In this case, smoothness and curvature, respectively, refer to the degree of roughness and the extent to which the surface bends, but despite their slightly different definitions, surface smoothness, and surface curvature are the same shape parameter but on different scales. With rougher and more curved surfaces, the existence of more convexities and concavities will make holes and objects appear earlier and prolong the number of iterations it takes for them to disappear (as a result of a full merge). See Fig. 3 for an example. See also supplementary note 1 for a synthetic experiment illustrating the relation between our suggested measures and shapes. The result of this experiment is summarized in Supplementary Fig. 1 and Supplementary Table 1.

To ensure numeric validity, the following filtering criteria were applied when performing statistics on the calculated max locations and FWHMs. For the average minimum distance between cristae across the matrix, we required the presence of cristae membrane and that there must exist different branches, folds, or instances of cristae membrane to measure against. In technical terms, we only included results computed from count curves with a maximum hole count ≥1. For crista width, we required that both the intracristal space volume and the cristae membrane volume be larger than 0.

Alternatives to persistent homology include methods that seek the largest contained sphere inside objects^56–58^ and where a larger sphere dominates neighboring and overlapping smaller spheres, and thus, statistics on object widths and internal distances will be biased towards larger values. The M-rep methodology represents 3-dimensional objects as 2-dimensional sheets defined by the collection of centers of spheres and their radii which has first or higher-order contact with the boundary in at least two places^59^. A calculation of the average widths can be done using Riemannian geometry on the 2-dimensional sheets. M-reps do not have the larger sphere bias but are considerably more complex to use than our method based on persistent homology.

### 2D to 3D relationships

Initially, the connected components of our mitochondria segmentation were used to extract the 3D sub-volume for each mitochondrion. Random image slices were then sampled from the nine predefined planes orientated at 45 degrees from each other to maximize data variability, see Fig. 2. The 2D perimeters and 2D cross-sectional areas of mitochondria were subsequently computed from the image slices and paired against their corresponding 3D surface areas and 3D volumes for further analysis. The comparison was made for varying sample sizes ranging from *n* = 1, which is equivalent to a direct single-data point comparison, to *n* = 100, where a comparison is made between the 2D and 3D averages of 100 data points. To ensure statistical reliability, 10,000 subsets of matching data points were randomly generated for each sample size tested.

Given a series of 2D parametric values and their 3D equivalents, we estimated the mapping function from 2D to 3D by first plotting the 2D values against the 3D values on a scatter plot, and then slided a window of size *ω* over the 2D value range on the x-axis. For each step of the moving window, we calculated the mean and std using the 3D values contained within the window. The mean is the expected 3D value for a given 2D value range and the std is the corresponding upper and lower bound: mean ± std. Since the parameters cannot be negative, all negative lower bounds were set to 0. To eliminate noise caused by data sparsity at the higher end of 2D values, we terminated the sliding window when it contained less than 10 data points. The size *ω* should be adjusted based on the scale of the 2D values, and we found empirically that $\omega =0.025* \max (\,{{\mbox{2D\_values}}}\,)$ works well.

The degree of correlation between 2D and 3D shape parameters can be summarized by computing the average error for approximated mapping functions:

7 $$\,{{\mbox{average}}}{{\mbox{\_}}}{{\mbox{error}}}\,=\frac{1}{T}* \mathop{\sum }\limits_{t=1}^{T}\left(\frac{\,{{\mbox{upper}}}{{\mbox{\_}}}{{{\mbox{bound}}}}_{t}+{{\mbox{lower}}}{{\mbox{\_}}}{{{\mbox{bound}}}}_{t}}{2* {{{\mbox{mean}}}}_{t}}\right)$$

where *T* is the total number of windows and *t* is the window index.

### Reporting summary

Further information on research design is available in the Nature Portfolio Reporting Summary linked to this article.

### Supplementary information

Supplementary Information

Description of Additional Supplementary Files

Supplementary Data 1

Reporting Summary

## Data Availability

The dataset is available from the website of École polytechnique fédérale de Lausanne (EPFL): https://www.epfl.ch/labs/cvlab/data/data-em/. A zip folder containing our cristae annotations can be found at: https://erda.ku.dk/workgroup/dikuUltrastructures/UCPH_IMAGE_cristae_dataset.zip. The source data behind Figs. 4 and 5 in the paper can be found in Supplementary Data 1. All other data are available from the corresponding author (or other applicable sources) on reasonable request.

## Code availability

The code for this paper can be found at:

•https://qim.dk/portfolio-items/3d-shape-analysis-using-persistent-homology/

•https://erda.ku.dk/workgroup/dikuUltrastructures/UCPH_IMAGE_persistent_homology.zip^63^ (alternative link)

The implementation is done in Python 3 with the help of the following major packages: TensorFlow 2/Keras, SciPy, NumPy, CC3D, OpenCV, Matplotlib, scikit-image and scikit-learn.

## References

[1] Underwood E, Redell JB, Zhao J, Moore AN, Dash PK (2020). A method for assessing tissue respiration in anatomically defined brain regions. Sci. Rep..

[2] Fried NT, Moffat C, Seifert EL, Oshinsky ML (2014). Functional mitochondrial analysis in acute brain sections from adult rats reveals mitochondrial dysfunction in a rat model of migraine. Am. J. Physiol. Cell Physiol..

[3] Cogliati S (2013). Mitochondrial cristae shape determines respiratory chain supercomplexes assembly and respiratory efficiency. Cell.

[4] Strauss M, Hofhaus G, Schroder RR, Kuhlbrandt W (2008). Dimer ribbons of atp synthase shape the inner mitochondrial membrane. EMBO J..

[5] Toth A (2020). Kinetic coupling of the respiratory chain with atp synthase, but not proton gradients, drives atp production in cristae membranes. Proc. Natl Acad. Sci. USA.

[6] Rieger B, Arroum T, Borowski MT, Villalta J, Busch KB (2021). Mitochondrial f(1) f(o) atp synthase determines the local proton motive force at cristae rims. EMBO Rep..

[7] Blum TB, Hahn A, Meier T, Davies KM, Kuhlbrandt W (2019). Dimers of mitochondrial ATP synthase induce membrane curvature and self-assemble into rows. Proc. Natl Acad. Sci. USA.

[8] Buzzard, E. et al. Cryo-electron tomography of C. elegans mitochondria reveals how the ATP synthase dimer interface shapes crista membranes. *bioRxiv* (2023). https://www.biorxiv.org/content/early/2023/02/06/2023.02.02.526626.full.pdf.

[9] Siegmund SE (2018). Three-dimensional analysis of mitochondrial crista ultrastructure in a patient with leigh syndrome by in situ cryoelectron tomography. iScience.

[10] Li X (2022). Ischemia-induced cleavage of opa1 at s1 site aggravates mitochondrial fragmentation and reperfusion injury in neurons. Cell Death Dis..

[11] Shi P (2022). Mechanical instability generated by myosin 19 contributes to mitochondria cristae architecture and oxphos. Nat. Commun..

[12] Yin X (2022). Sam50 exerts neuroprotection by maintaining the mitochondrial structure during experimental cerebral ischemia/reperfusion injury in rats. CNS Neurosci. Ther.

[13] Perkins G (1997). Electron tomography of neuronal mitochondria: three-dimensional structure and organization of cristae and membrane contacts. J. Struct. Biol..

[14] Perkins GA (1997). Electron tomography of large, multicomponent biological structures. J. Struct. Biol..

[15] Mendelsohn R (2022). Morphological principles of neuronal mitochondria. J. Comp. Neurol..

[16] Cserep, C., Posfai, B., Schwarcz, A. D. & Denes, A. Mitochondrial ultrastructure is coupled to synaptic performance at axonal release sites. *eNeuro* **5** (2018). https://www.ncbi.nlm.nih.gov/pubmed/29383328. 10.1523/ENEURO.0390-17.2018 PMC5788698 29383328

[17] Vincent AE (2019). Quantitative 3d mapping of the human skeletal muscle mitochondrial network. Cell Rep..

[18] Hu C (2020). Opa1 and micos regulate mitochondrial crista dynamics and formation. Cell Death Dis..

[19] Huang X (2018). Fast, long-term, super-resolution imaging with hessian structured illumination microscopy. Nat. Biotechnol..

[20] Segawa, M. et al. Quantification of cristae architecture reveals time-dependent characteristics of individual mitochondria. *Life Sci. Alliance* **3**, e201900620 (2020). 10.26508/lsa.201900620 PMC7283135 32499316

[21] Wang C (2019). A photostable fluorescent marker for the superresolution live imaging of the dynamic structure of the mitochondrial cristae. Proc. Natl Acad. Sci. USA.

[22] Xiao C (2018). Automatic mitochondria segmentation for em data using a 3d supervised convolutional network. Front Neuroanat..

[23] Liu J (2020). Automatic reconstruction of mitochondria and endoplasmic reticulum in electron microscopy volumes by deep learning. Front Neurosci..

[24] Perslev, M., Dam, E. B., Pai, A. & Igel, C. One network to segment them all: A general, lightweight system for accurate 3d medical image segmentation. In *MICCAI 2019, LNCS*, Vol. 11765 (eds Shen, D. et al.) 30–38 (Springer International Publishing, 2019).

[25] Ronneberger, O., Fischer, P. & Brox, T. U-net: Convolutional networks for biomedical image segmentation. In *MICCAI 2015, Part III, LNCS*, Vol. 9351, 234–241 (Springer Verlag, 2015).

[26] Zomorodian A, Carlsson G (2005). Computing persistent homology. Discrete Comput. Geometry.

[27] Gomes LC, Di Benedetto G, Scorrano L (2011). During autophagy mitochondria elongate, are spared from degradation and sustain cell viability. Nat. Cell Biol..

[28] Patten DA (2014). Opa1-dependent cristae modulation is essential for cellular adaptation to metabolic demand. EMBO J..

[29] Scorrano L (2002). A distinct pathway remodels mitochondrial cristae and mobilizes cytochrome c during apoptosis. Dev. Cell.

[30] Frezza C (2006). Opa1 controls apoptotic cristae remodeling independently from mitochondrial fusion. Cell.

[31] Rajab BS (2022). Differential remodelling of mitochondrial subpopulations and mitochondrial dysfunction are a feature of early stage diabetes. Sci. Rep..

[32] Twig G (2008). Fission and selective fusion govern mitochondrial segregation and elimination by autophagy. EMBO J..

[33] Rambold AS, Kostelecky B, Elia N, Lippincott-Schwartz J (2011). Tubular network formation protects mitochondria from autophagosomal degradation during nutrient starvation. Proc. Natl Acad. Sci. USA.

[34] Frank S (2001). The role of dynamin-related protein 1, a mediator of mitochondrial fission, in apoptosis. Dev. Cell.

[35] Trevisan T (2018). Manipulation of mitochondria dynamics reveals separate roles for form and function in mitochondria distribution. Cell Rep..

[36] Hasselholt S, Hahn U, Vedel Jensen EB, Nyengaard JR (2019). Practical implementation of the planar and spatial rotator in a complex tissue: the brain. J. Microsc..

[37] Laprade, W. M., Perslev, M. & Sporring, J. How few annotations are needed for segmentation using a multi-planar u-net? In *Deep Generative Models, and Data Augmentation, Labelling, and Imperfections, LNCS*, Vol. 13003 (eds Engelhardt, S. et al.), 209–216 (Springer International Publishing, Cham, 2021).

[38] Stephan T, Roesch A, Riedel D, Jakobs S (2019). Live-cell sted nanoscopy of mitochondrial cristae. Sci. Rep..

[39] Vincent AE (2016). The spectrum of mitochondrial ultrastructural defects in mitochondrial myopathy. Sci. Rep..

[40] Plecita-Hlavata L (2016). Hypoxic hepg2 cell adaptation decreases atp synthase dimers and atp production in inflated cristae by mitofilin down-regulation concomitant to micos clustering. FASEB J..

[41] Grieco JP (2020). Progression-mediated changes in mitochondrial morphology promotes adaptation to hypoxic peritoneal conditions in serous ovarian cancer. Front Oncol..

[42] Han M (2023). Spatial mapping of mitochondrial networks and bioenergetics in lung cancer. Nature.

[43] Cali C (2018). The effects of aging on neuropil structure in mouse somatosensory cortex-a 3d electron microscopy analysis of layer 1. PLoS ONE.

[44] Miranda-Astudillo H, Ostolga-Chavarria M, Cardol P, Gonzalez-Halphen D (2022). Beyond being an energy supplier, ATP synthase is a sculptor of mitochondrial cristae. Biochim. Biophys. Acta Bioenerg..

[45] Panek T, Elias M, Vancova M, Lukes J, Hashimi H (2020). Returning to the fold for lessons in mitochondrial crista diversity and evolution. Curr. Biol..

[46] Brandt T (2017). Changes of mitochondrial ultrastructure and function during ageing in mice and *Drosophila*. eLife.

[47] Smith A (2022). Rootpainter: deep learning segmentation of biological images with corrective annotation. New Phytologist.

[48] Kuo, W., Häne, C., Yuh, E., Mukherjee, P. & Malik, J. Cost-sensitive active learning for intracranial hemorrhage detection. In *Medical Image Computing and Computer Assisted Intervention – MICCAI 2018, LNCS*, Vol. 11072, (eds Frangi, A. F., Schnabel, J. A., Davatzikos, C., Alberola-López, C. & Fichtinger, G.) 715–723 (Springer International Publishing, Cham, 2018).

[49] Knott, G. & Cantoni, M. Electron microscopy dataset (2013) https://cvlab.epfl.ch/data/data-em/. Accessed: 2020-03-14.

[50] Stephensen HJT, Darkner S, Sporring J (2020). Restoring drifted electron microscope volumes using synaptic vesicles at sub-pixel accuracy. Commun. Biol..

[51] Kaji, S., Sudo, T. & Ahara, K. Cubical ripser: Software for computing persistent homology of image and volume data (2020). https://arxiv.org/abs/2005.12692.

[52] Osher S, Sethian JA (1988). Fronts propagating with curvature-dependent speed: algorithms based on hamilton-jacobi formulations. J. Comput. Phys..

[53] Sethian, J. A. Level Set Methods and Fast Marching Methods. In *Cambridge Monograph on Applied and Computational Mathematics*, 2nd Ed., 1–164 (Cambridge University Press, 1999).

[54] Osher, S. & Fedki, R. Level Set Methods and Dynamic Implicit Surfaces. In *Applied Mathematical Sciences*, Vol. 153, 3–93 (Springer New York, NY, 2002).

[55] Breuss M, Weickert J (2006). A shock-capturing algorithm for the differential equations of dilation and erosion. J. Math Imaging Vis..

[56] Dahl, V. A. & Dahl, A. B. Fast local thickness. In *Proceedings of the IEEE/CVF Conference on Computer Vision and Pattern Recognition Workshops*. 4336–4344 (2023).

[57] Gostick J (2019). Porespy: A python toolkit for quantitative analysis of porous media images. J. Open Source Softw..

[58] Dougherty R, Kunzelmann K-H (2007). Computing local thickness of 3d structures with imagej. Microscopy Microanal..

[59] Pizer S (2003). Deformable m-reps for 3d medical image segmentation. Int. J. Comput. Vis..

[60] Gilkerson RW, Selker JM, Capaldi RA (2003). The cristal membrane of mitochondria is the principal site of oxidative phosphorylation. FEBS Lett..

[61] Vogel F, Bornhovd C, Neupert W, Reichert AS (2006). Dynamic subcompartmentalization of the mitochondrial inner membrane. J. Cell Biol..

[62] Giacomello M, Pyakurel A, Glytsou C, Scorrano L (2020). The cell biology of mitochondrial membrane dynamics. Nat. Rev. Mol. Cell Biol..

[63] Wang, C., Østergaard, L., Hasselholt, S. & Sporring, J. Link to software and dataset used in this paper. (2024) 10.17894/ucph.7ee0fe29-2f56-43ef-913e-bafce56c1134

