# Supplementary material for: A semi-automatic method for extracting mitochondrial cristae characteristics from 3D focused ion beam scanning electron microscopy data
Source: Commun Biol. 2024 Mar 28;7:377. doi: 10.1038/s42003-024-06045-4 (PMC10978844; doi:10.1038/s42003-024-06045-4)
Supplement: Supplementary file 1 — Supplementary Information [file 42003_2024_6045_MOESM1_ESM.pdf]

# Supplementary Information

## Supplementary Note 1 Simplified Persistent Homology Example

To demonstrate how the max count location and FWHM can be used as shape measures, we generate sample images of size $(n, m)$ in 2D using the equation:

$$f(x) \sim \mathcal{N}(\mu_1,\, \sigma_1^2) \tag{1}$$

$$g(x) \sim \mathcal{N}(\mu_2,\, \sigma_2^2) \tag{2}$$

$$h(x) = \frac{1}{\sqrt{2\pi}\sigma_3} e^{-\frac{x^2}{2\sigma_3^2}} \tag{3}$$

$$I(x, y) = \begin{cases} 0, & \text{if } (f * h)(x) < y < (g * h)(x) \\ 1, & \text{else} \end{cases} \tag{4}$$

The idea here is to use 2 normal distributed and smoothed sequences, $f * h$ and $g * h$, to represent neighboring cristae membranes that we would like to measure. In our equation, $\mu_1$ and $\mu_2$ are the means of the Gaussian distribution (where $\mu_1 < \mu_2$), and they correspond to the theoretical row-wise locations of the membranes within our sample image I; $\sigma_1$ and $\sigma_2$ are the standard deviations of each sequence, which specifies the degree of roughness/curvature of the generated membranes; and finally, $\sigma_3$ specifies the strength of the Gaussian smoothing filter, which is needed to reduce excessive local raggedness.

In our experiment, we generated 3 datasets of 1000 images, where each dataset has its own unique theoretical distance $(\mu_1 - \mu_2)$ between the membranes and unique levels of roughness/curvature (see supplementary fig. 1).

**Supplementary Figure 1:** Examples of the generated images.

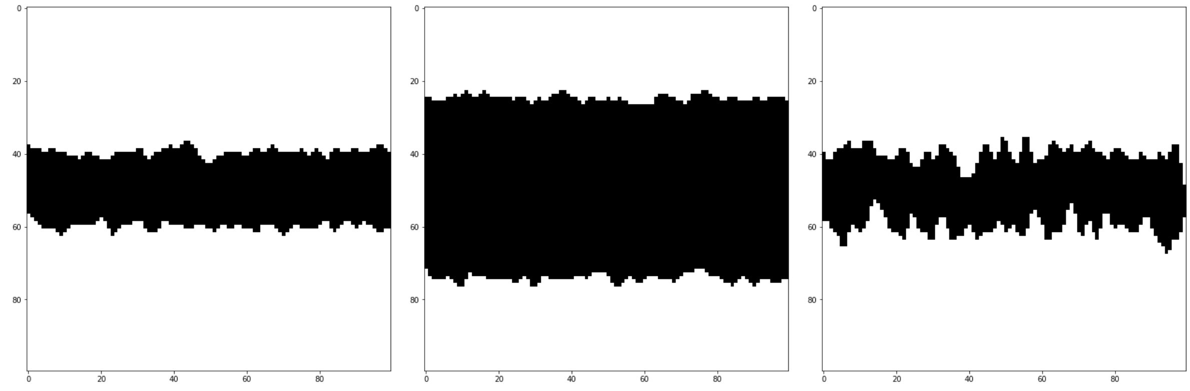

**(a)** **(b)** **(c)**

Image examples from (a) dataset 1 with $\mu_1 = 40$, $\mu_2 = 60$, $\sigma_1 = \sigma_2 = 2$, $\sigma_3 = 1$, (b) dataset 2 with $\mu_1 = 25$, $\mu_2 = 75$, $\sigma_1 = \sigma_2 = 2$, $\sigma_3 = 1$, (c) dataset 3 with $\mu_1 = 40$, $\mu_2 = 60$, $\sigma_1 = \sigma_2 = 5$, $\sigma_3 = 1$

As the region which we would like to measure is black in the images, the dilation and hole-counting version of persistent homology is used. The measured results are shown in supplementary table 1. We see that the max location correlates with the distance between the synthetic membranes, and the FWHM correlates with the roughness of the membrane surfaces.

**Supplementary Table 1:** Persistent homology results on artificially generated datasets from supplementary fig. 1. Column 1 shows the true half distances to be compared directly against the measured half distances/max locations in column 3. These generally correlate quite well, but there is a small degree of underestimation in the half distances. Column 2 records the standard deviations used for generating the curves, and is to be used for indirect comparisons against measured FWHM in column 4. The values in columns 2 and 4 are not supposed to match, but they should have the same in-column ordering in terms of value size, which is the case here. FWHM; full-width-half-maximum.

| Experiement | $(\mu_2 - \mu_1)/2$ | $\sigma_1 = \sigma_2$ | max location | FWHM |
|---|---|---|---|---|
| 1 | 10 | 2 | 9.43 | 1.79 |
| 2 | 25 | 2 | 24.41 | 1.77 |
| 3 | 10 | 5 | 8.13 | 3.02 |

# Supplementary Note 2 Surface Area/Volume Ratio Is Unsuitable as a Shape Descriptor

In the following simple example, we try to illustrate the challenge in using the ratio between surface area and volume as a shape descriptor for mitochondrial cristae. A tubular shape of length $L$ for large values of $L$ and a circular cross-section of radius $r$ (corresponding to a type of cylindrical crista) has approximate volume $\pi r^2 L$, surface area $2\pi r L$, and surface area/volume ratio $\frac{2}{r}$. In contrast, another tubular shape of the same length $L$ but with a rectangular cross-section with side-lengths $a$ and $b$ (corresponding to a more lamellar crista) has approximate volume $abL$, surface area $(2a + 2b)L$, and surface area/volume ratio $\frac{2(a+b)}{ab}$. Therefore, any given surface area/volume ratio value $k$ can correspond to either of the two shapes. Further, assuming circular tubes, we can find the radius as $\frac{2}{k}$, but assuming rectangular tubes with $a \geq b$, choosing any value for $b > \frac{2}{k}$, then $a = \frac{2b}{bk-2}$, and with $a \geq b$ we can infer that $b$ must also be limited from above as, $\frac{4}{k} \geq b > \frac{2}{k}$. Thus, choosing $b = \frac{4}{k} \Rightarrow a = \frac{4}{k}$ and choosing $b \to \frac{2}{k} \Rightarrow a \to \infty$.

# Supplementary Note 3 Segmentation Performance

**Supplementary Table 2:** Performance of the segmentation model used in this study. For the mitochondria, a 3D segmentation was produced by the 2D multiplanar UNet and then compared against the 3D ground truth. The segmentations of the crista membrane and intracristal space are evaluated in 2D (without the multi-planar aspect) because the 3D volume is not fully annotated for cristae and intracristal space.

| Parameter | F1 score (%) | Sensitivity (%) | Specificity (%) |
|---|---|---|---|
| Mitochondrion | 0.929 | 0.916 | 0.997 |
| Crista membrane | 0.582 | 0.681 | 0.991 |
| Intracristal space | 0.643 | 0.600 | 0.997 |

The mathematical definitions for the pixel-based performance metrics used in supplementary table 2 are provided below.

$$F_1 = \frac{2 * \text{True Positive}}{2 * \text{True Positive} + \text{False Positive} + \text{False Negative}} \quad (5)$$

$$\text{Recall} = \text{Sensitivity} = \frac{\text{True Positive}}{\text{True Positive} + \text{False Negative}} \quad (6)$$

$$\text{Specificity} = \frac{\text{True Negative}}{\text{True Negative} + \text{False Positive}} \quad (7)$$

The standard metrics, F1 score and Sensitivity, are not the most appropriate for crista membrane and intracristal space, because their structures are extremely thin and long, meaning that a slight mismatch will produce a large error value. Since it is impossible for manual annotation to be perfectly consistent at deciding the exact shade of grey to include, we propose an alternative assessment, where dilation is applied on the segmentation result and the sensitivity is recorded at each step. Since one round of dilation adds a single layer of voxels around the segmentation, a large increase in sensitivity within the first iteration implies the segmentation is good, despite the low scores reported in supplementary table 2.

**Supplementary Figure 2:** Results of the dilation-sensitivity tests.

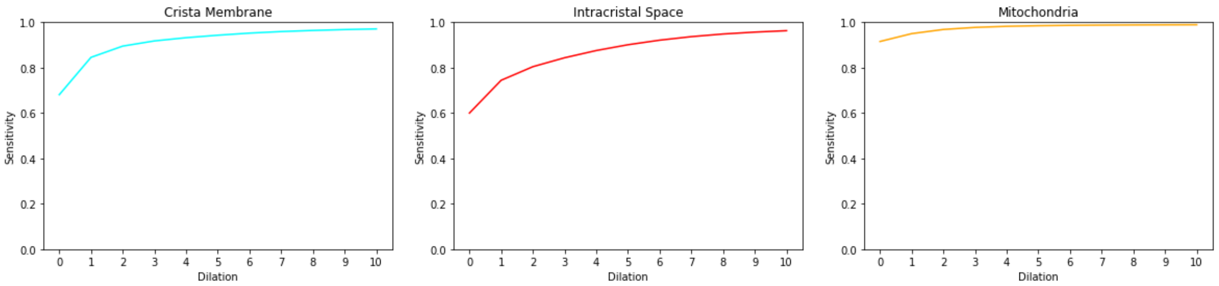


**(a)** **(b)** **(c)**

Results of the dilation-sensitivity test for crista membrane (a), intracristal space (b) and mitochondria (c). The effect of dilation on sensitivity is much greater on crista membrane and intracristal space than mitochondria, where they jumped from 0.681 to 0.846 and 0.600 to 0.745 respectively. By dilation round 2, crista membrane reached 0.895 and intracristal space reached 0.804. This experiment shows that the segmentation results are in fact quite close to the manual annotations

The model's ability to separate mitochondria is also tested by performing connected components on both the segmentation result and the manually labelled test set (which is one of the small subvolumes provided by the dataset). Using a minimum volume criterion of $25^3$ voxel$^3$ (same as our main paper), 28 mitochondria are counted in our segmentation and 29 mitochondria are counted in the test set. The count difference of 1 could be caused by an incorrect merging of mitochondria, but it may also be something else (e.g. the model under-segmented a single mitochondrion and it fell under the minimum volume). This indicates that our model can separate mitochondria to the same degree as what is possible with manual annotation in the current dataset.

**Supplementary Figure 3:** Comparison of 2D and 3D measurements.

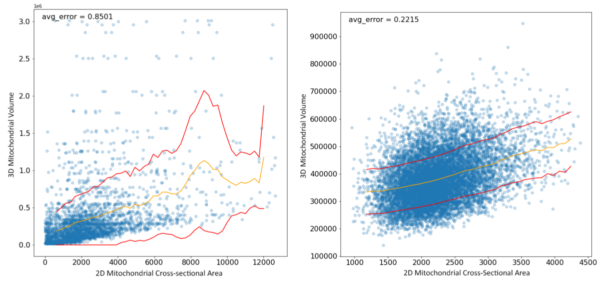


**(a)** mito_vol: n = 1 **(b)** mito_vol: n = 25 **(c)** mito_vol: n = 50 **(d)** mito_vol: n = 100

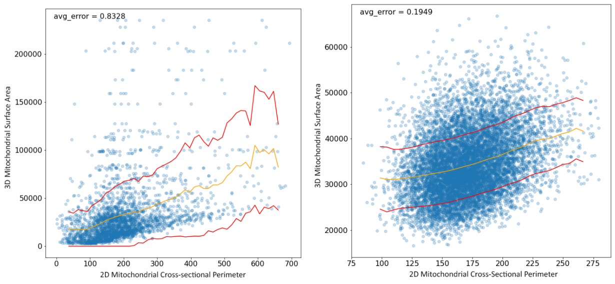


**(e)** mito_surface: n = 1 **(f)** mito_surface: n = 25 **(g)** mito_surface: n = 50 **(h)** mito_surface: n = 100

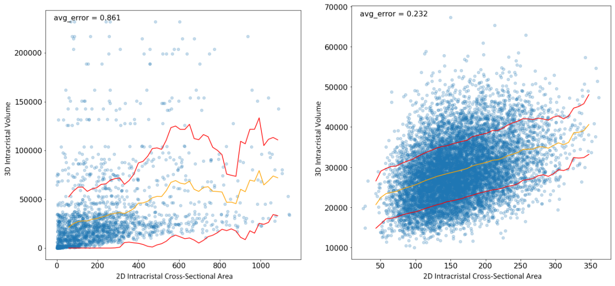


**(i)** intracristal_vol: n = 1 **(j)** intracristal_vol: n = 25 **(k)** intracristal_vol: n = 50 **(l)** intracristal_vol: n = 100

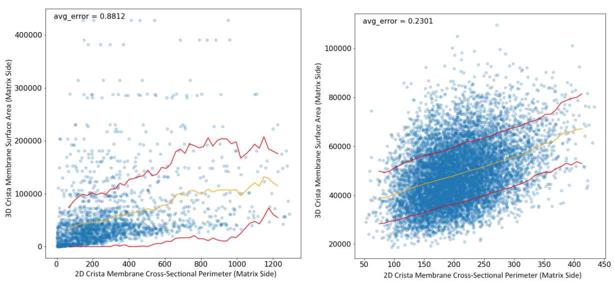


**(m)** cristae_surface: n = 1 **(n)** cristae_surface: n = 25 **(o)** cristae_surface: n = 50 **(p)** cristae_surface: n = 100

Shape parameters in 2D are measured using image slices from random planes and random slice numbers in the structure of interest and compared against their 3D equivalents in random subsets of size n = 1 to n = 100. The comparison is done in terms of subset means and is plotted in a scatter plot. To estimate the mapping function from 2D to 3D, a sliding window is applied along the 2D value range on the x-axis, where at each step, we calculate the mean and standard deviation using the 3D values contained within the window. The mean acts as the expected 3D value for a given 2D value range and the standard deviation acts as its upper and lower bound. The function curves are respectively overlaid on top of the scatter plot using yellow and red.
