# Supplementary material for: A semi-automatic method for extracting mitochondrial cristae characteristics from 3D focused ion beam scanning electron microscopy data
Source: Commun Biol. 2024 Mar 28;7:377. doi: 10.1038/s42003-024-06045-4 (PMC10978844; doi:10.1038/s42003-024-06045-4)
Supplement: Supplementary file 2 — Description of Additional Supplementary Files [file 42003_2024_6045_MOESM2_ESM.pdf]

Description of Additional Supplementary Files

File name: Supplementary Data 1

Description: The source data behind the figs 4 - 5 in the paper.
