# Supplementary material for: A semi-automatic method for extracting mitochondrial cristae characteristics from 3D focused ion beam scanning electron microscopy data
Source: Commun Biol. 2024 Mar 28;7:377. doi: 10.1038/s42003-024-06045-4 (PMC10978844; doi:10.1038/s42003-024-06045-4)
Supplement: Supplementary file 4 — Reporting Summary [file 42003_2024_6045_MOESM4_ESM.pdf]

nature portfolio

Corresponding author(s): Jon Sporring, Chenhao Wang

Last updated by author(s): Jan 26, 2024

Reporting Summary

Nature Portfolio wishes to improve the reproducibility of the work that we publish. This form provides structure for consistency and transparency in reporting. For further information on Nature Portfolio policies, see our Editorial Policies and the Editorial Policy Checklist.

Statistics

For all statistical analyses, confirm that the following items are present in the figure legend, table legend, main text, or Methods section.

n/a	Confirmed	
☐	☒	The exact sample size (*n*) for each experimental group/condition, given as a discrete number and unit of measurement
☐	☒	A statement on whether measurements were taken from distinct samples or whether the same sample was measured repeatedly
☐	☒	The statistical test(s) used AND whether they are one- or two-sided<br>*Only common tests should be described solely by name; describe more complex techniques in the Methods section.*
☐	☒	A description of all covariates tested
☐	☒	A description of any assumptions or corrections, such as tests of normality and adjustment for multiple comparisons
☐	☒	A full description of the statistical parameters including central tendency (e.g. means) or other basic estimates (e.g. regression coefficient) AND variation (e.g. standard deviation) or associated estimates of uncertainty (e.g. confidence intervals)
☐	☒	For null hypothesis testing, the test statistic (e.g. *F*, *t*, *r*) with confidence intervals, effect sizes, degrees of freedom and *P* value noted<br>*Give P values as exact values whenever suitable.*
☒	☐	For Bayesian analysis, information on the choice of priors and Markov chain Monte Carlo settings
☒	☐	For hierarchical and complex designs, identification of the appropriate level for tests and full reporting of outcomes
☐	☒	Estimates of effect sizes (e.g. Cohen's *d*, Pearson's *r*), indicating how they were calculated

Our web collection on statistics for biologists contains articles on many of the points above.

Software and code

Policy information about availability of computer code

Data collection	The raw 3D image data were acquired by Graham Knott and Marco Cantoni from École polytechnique fédérale de Lausanne (see Data). Matlab R2022a used for image registration to align the raw images, before image analysis. Photoshop (2021 and 2022) used to produce the manual annotation of cristae and intracristal space, which forms the training data for segmentation model implemented in tensorflow. (see below)
Data analysis	We implemented our custom code in Python 3 with packages numpy, scipy, opencv, skimage, matplotlib, tensorflow, keras, cc3d, imutils, tqdm. The code is available for editors and reviewers upon request and will be made available after publication at https://erda.ku.dk/workgroup/dikuUltrastructures/UCPH_IMAGE_persistent_homology.zip

For manuscripts utilizing custom algorithms or software that are central to the research but not yet described in published literature, software must be made available to editors and reviewers. We strongly encourage code deposition in a community repository (e.g. GitHub). See the Nature Portfolio guidelines for submitting code & software for further information.

Data

Policy information about availability of data

All manuscripts must include a data availability statement. This statement should provide the following information, where applicable:

- Accession codes, unique identifiers, or web links for publicly available datasets
- A description of any restrictions on data availability
- For clinical datasets or third party data, please ensure that the statement adheres to our policy

Full version of our statement can be found in "6. Data availability" in our manuscript. The Lausanne dataset is available from the website of Ecole polytechnique federale de Lausanne (EPFL): https://www.epfl.ch/labs/cvlab/data/data-em/
A zip folder containing our cristae annotations can be found at: https://erda.ku.dk/workgroup/dikuUltrastructures/UCPH_IMAGE_cristae_dataset.zip.

Research involving human participants, their data, or biological material

Policy information about studies with human participants or human data. See also policy information about sex, gender (identity/presentation), and sexual orientation and race, ethnicity and racism.

Reporting on sex and gender	Our research does not involve human participants, their data, or biological material.
Reporting on race, ethnicity, or other socially relevant groupings	Our research does not involve human participants, their data, or biological material.
Population characteristics	Our research does not involve human participants, their data, or biological material.
Recruitment	Our research does not involve human participants, their data, or biological material.
Ethics oversight	Our research does not involve human participants, their data, or biological material.

Note that full information on the approval of the study protocol must also be provided in the manuscript.

Field-specific reporting

Please select the one below that is the best fit for your research. If you are not sure, read the appropriate sections before making your selection.

☒ Life sciences ☐ Behavioural & social sciences ☐ Ecological, evolutionary & environmental sciences

For a reference copy of the document with all sections, see nature.com/documents/nr-reporting-summary-flat.pdf

Life sciences study design

All studies must disclose on these points even when the disclosure is negative.

Sample size	A total of 496 mitochondria and their internal components were segmented from the raw image volume.
Data exclusions	The segmentation output was filtered.<br><br>Mitochondria segmentations with a volume of less than 25^3 voxels^3 were excluded. Furthermore, mitochondria touching the boundary of the image volume, impairing analysis of the complete organelle, were excluded.<br><br>Additional parameter-dependent filtering was performed. For example, when measuring cristae membrane surface area, the cristae membrane should be visible/detectable within the specific mitochondrion. Please refer to the manuscript for details on filtering for individual parameters. Sample sizes are stated on all individual plots.
Replication	Since all measurements are computer based, the same code on the same data will always produce the same results. For analyses using a random subset of the total dataset (mostly related to 2D to 3D correlation), the random subset sampling was repeated tens of thousands times to ensure statistical reliability.
Randomization	Generally, all the data points available within the image volume were included in the analyses. For the few tests that involve sampling, the samples were random (either from uniform distribution or normal distribution).
Blinding	Not applicable.

Reporting for specific materials, systems and methods

We require information from authors about some types of materials, experimental systems and methods used in many studies. Here, indicate whether each material, system or method listed is relevant to your study. If you are not sure if a list item applies to your research, read the appropriate section before selecting a response.

Materials & experimental systems

n/a	Involved in the study
☒	☐ Antibodies
☒	☐ Eukaryotic cell lines
☒	☐ Palaeontology and archaeology
☒	☐ Animals and other organisms
☒	☐ Clinical data
☒	☐ Dual use research of concern
☒	☐ Plants

Methods

n/a	Involved in the study
☒	☐ ChIP-seq
☒	☐ Flow cytometry
☒	☐ MRI-based neuroimaging
